# IRES-mediated cap-independent translation, a path leading to hidden proteome

**DOI:** 10.1093/jmcb/mjz091

**Published:** 2019-09-03

**Authors:** Yun Yang, Zefeng Wang

**Affiliations:** 1 CAS Key Laboratory of Computational Biology, Biomedical Big Data Center, CAS-MPG Partner Institute for Computational Biology, Shanghai Institute of Nutrition and Health, Chinese Academy of Sciences, Shanghai 200031, China; 2 University of Chinese Academy of Sciences, Chinese Academy of Sciences, Shanghai 200031, China; 3 CAS Center for Excellence in Molecular Cell Science, Chinese Academy of Sciences, Shanghai 200031, China

**Keywords:** IRES, ITAF, translation, bicistronic system, circular RNA

## Abstract

Most eukaryotic mRNAs are translated in a cap-dependent fashion; however, under stress conditions, the cap-independent translation driven by internal ribosomal entry sites (IRESs) can serve as an alternative mechanism for protein production. Many IRESs have been discovered from viral or cellular mRNAs to promote ribosome assembly and initiate translation by recruiting different *trans*-acting factors. Although the mechanisms of translation initiation driven by viral IRESs are relatively well understood, the existence of cellular IRESs is still under debate due to the limitations of translation reporter systems used to assay IRES activities. A recent screen identified > 1000 putative IRESs from viral and human mRNAs, expanding the scope and mechanism for cap-independent translation. Additionally, a large number of circular RNAs lacking free ends were identified in eukaryotic cells, many of which are found to be translated through IRESs. These findings suggest that IRESs may play a previously unappreciated role in driving translation of the new type of mRNA, implying a hidden proteome produced from cap-independent translation.

## Introduction

In eukaryotes, a m7G cap is added to the 5′ end of most precursor messenger RNAs (pre-mRNAs) during RNA synthesis (Moteki and Price, 2002). This cap structure can be recognized by the eukaryotic initiation factor 4E (eIF4E), a component of the eIF4F complex consisting of eIF4E, eIF4G, and eIF4A. The eIF4F complex further facilitates the recruitment of the pre-assembled 43S pre-initiation complex (PIC) that includes the 40S small ribosomal subunit, eIF1, eIF1A, eIF3, eIF5, and the eIF2/Met-tRNAi/GTP ternary complex. Following assembly, the 43S PIC complex scans the 5′ untranslated region (5′ UTR) of the mRNAs in a 5′ to 3′ direction to reach the start codon (typically AUG) and subsequently employs 60S large ribosomal subunit to form an 80S ribosome to initiate peptide synthesis (for a mechanistic review, see Sonenberg and Hinnebusch, 2009). This process is known as the cap-dependent translation initiation, which is a primary mode of translation initiation in eukaryotic cells.

Under certain conditions, mRNAs cannot be translated through the cap-dependent translation. For example, the cap-dependent translation is inhibited under cellular stress and viral infection, and some viral mRNAs that are efficiently translated in their host cells do not even contain a 5′ cap structure (Kneller et al., 2006; Spriggs et al., 2010; Stern-Ginossar et al., 2018). In such cases, an alternative mechanism known as cap-independent translation is often used to initiate mRNA translation through the internal ribosome entry site (IRES). The endogenous genes capable of cap-independent translation are usually involved in biological pathways in response to cellular stress or viral infection, implying that IRES-mediated cap-independent translation plays an important role under such cellular conditions. Alternatively, the protein translation under stress conditions can also be regulated through upstream open reading frames (ORFs) within the 5′ UTRs. Typically, upstream ORFs suppress translation of their associated downstream coding regions under normal growth condition. However, under cellular stresses, the suppression by upstream ORFs is released and thus translation of the downstream ORFs can be activated/increased (Andreev et al., 2015; Young and Wek, 2016). In this review, we will focus on the mechanism of the IRES-driven cap-independent translation and the experimental systems that are used to study them. The related topics such as biological functions of cap-independent translation and regulation through upstream ORFs can be found in other excellent reviews (Spriggs et al., 2010; Komar and Hatzoglou, 2011; Lacerda et al., 2017).

## Internal ribosome entry sites in the viral genome

**Figure 1:**
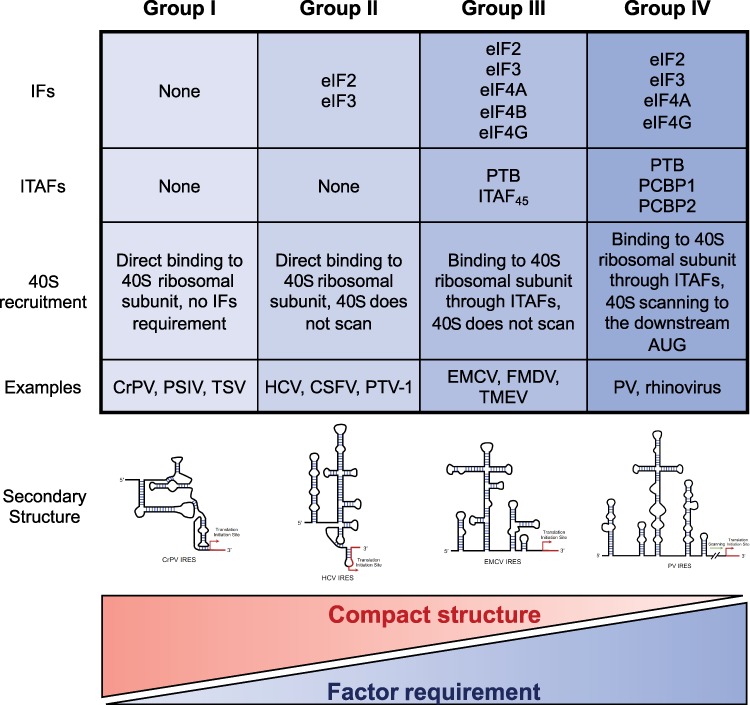
Four groups of viral IRESs. Viral IRESs can be classified into four groups based on their structures and the requirement of ITAFs and IFs. Generally, IRESs with more compact structures require less auxiliary protein factors.

By definition, IRESs are the RNA elements that recruit ribosomes to the internal region of mRNAs to initiate translation through a cap-independent pathway. They were originally discovered in the viruses of the *Picornaviridae* family such as poliovirus (PV) and encephalomyocarditis virus (Jang et al., 1988; Pelletier and Sonenberg, 1988). A large number of IRESs were later identified in pathogenic viruses, including human immunodeficiency virus, hepatitis C virus (HCV), and foot and mouth disease virus (Belsham and Brangwyn, 1990; Tsukiyama-Kohara et al., 1992; Buck et al., 2001). Although these viral IRESs contain diverse sequences, many of them have similar secondary structures and initiate translation through similar mechanisms. In addition, the activities of IRESs often require assistance from other factors known as IRES-transacting factors (ITAFs). Based on the structures and the requirement of translation initiation factors (IFs) and ITAFs, the viral IRESs are classified into four groups (Figure 1; Kieft, 2008). Generally, IRESs with more tightly folded RNA structures require fewer protein factors (including IFs and ITAFs) to promote translation initiation; however, the mechanism behind this seemingly compensatory relationship is unclear.

The Group I viral IRESs generally have strong activities and can initiate translation from a non-AUG start codon without additional ITAFs or even eIF2/Met-tRNAi/GTP ternary complex. These IRESs are folded to a compact structure that includes three pseudoknots (PKI, PKII, and PKIII) and multiple stem-loops to directly interact with the 40S small ribosomal subunit (Kanamori and Nakashima, 2001; Jan and Sarnow, 2002; Nishiyama et al., 2003). The structures of Group II IRESs in ribosome complex reveals that the PKI pseudoknot mimics a codon-anticodon interaction between an mRNA and tRNA and can be loaded into the A site of ribosome without initiator Met-tRNAi (Spahn et al., 2004; Costantino et al., 2008; Muhs et al., 2015). Interestingly, the CrPV IRES can initiate translation by interacting with bacterial ribosomes using the tRNA mimicry, suggesting a similarity of translation initiation between eukaryotes and bacteria (Colussi et al., 2015).

The Group II IRESs can also directly interact with 40S small ribosomal subunit with specialized RNA structure, but their activities usually require assistance of several IFs including eIF2 and eIF3 and initiator Met-tRNAi (Tsukiyama-Kohara et al., 1992; Rijnbrand et al., 1997; Pisarev et al., 2004; Figure 1). For example, the HCV IRES contains three structural domains that interact with 40S small ribosomal subunit. The Domain II binds to the 40S small ribosomal subunit at the E site (Spahn et al., 2001), and the domain IIId paired with 18S rRNA to stabilize the interaction between IRES and 40S small ribosomal subunit (Matsuda and Mauro, 2014). More details on the structures and the interaction between IRES and ribosome can be found in the in-depth review by others (Kieft, 2008).

The other two groups of viral IRESs, Group III and Group IV, cannot bind to the 40S small ribosomal subunit directly. Instead, they recruit the 40S small ribosomal subunit through different ITAFs and require canonical IFs in the cap-dependent translation (i.e. eIF2, eIF3, eIF4A, eIF4B, and eIF4G) (Pestova et al., 1996a; Pestova et al., 1996b; Lomakin et al., 2000). The major difference between Group III and Group IV IRESs is the requirement of 40S ribosome scanning. In the *in vitro* translation systems, Group III IRESs usually initiate translation at the 40S subunit recruitment site and thus 40S ribosome scanning is unnecessary (Pestova et al., 1996a), whereas the 40S ribosome scans the untranslated region in a 5′ to 3′ direction to reach the downstream AUG start site in the Group IV IRES-mediated translation initiation (Sweeney et al., 2014). However, such distinction may change when the IRES activities are measured using *in vivo* systems: some Group IV IRESs (e.g. EV7, PV1, and EV-A71) were recently reported to initiate translation from the upstream ORF's start codon to produce the UP protein without 40S ribosome scanning during viral infection (Lulla et al., 2019). Since translation initiation by IRESs of these two groups shares many common features with the canonical translation, they are probably affected by the reagents that inhibit cap-dependent translation of mRNA.

## Internal ribosome entry sites in the eukaryotic genome

In addition to the viral mRNA, IRESs were also found in cellular mRNAs, many of which encode proteins required in stress response, e.g. in conditions of apoptosis, mitosis, hypoxia, and nutrient limitation (reviewed in Komar and Hatzoglou, 2011). Generally, cellular IRESs contain fewer RNA structures compared to the viral IRESs and share little sequence conservation among them, and thus are difficult to be classified into different groups. The lack of sequence/structure similarity in cellular IRESs also makes it difficult to predict novel endogenous IRESs in mRNAs. On the other hand, the large diversity of endogenous IRESs in eukaryotic mRNAs also suggests that more diverse and complicate mechanisms may be used by endogenous IRESs to drive cap-independent translation.

**Figure 2:**
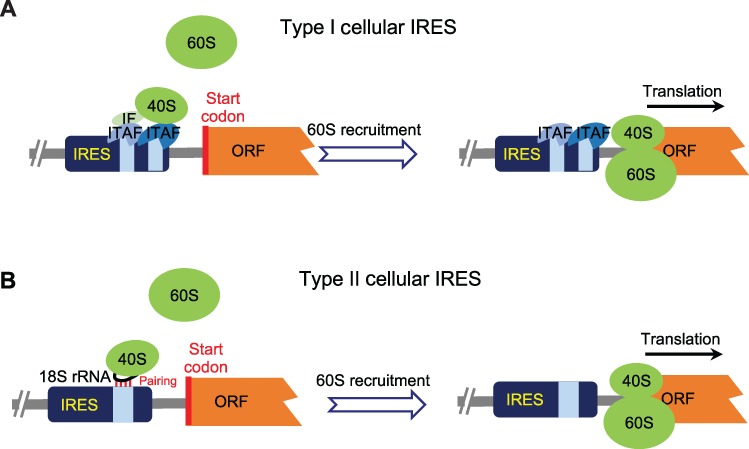
Mechanism of translation initiation driven by cellular IRES. (**A**) Type I cellular IRES. The *cis*-elements in the cellular IRESs are bound by the ITAFs. These ITAFs will directly interact with the 40S ribosomal subunit or recruit the 40S ribosomal subunit through the ‘bridge’—IFs. Finally, the 60S ribosomal subunit is recruited to start translation. (**B**) Type II cellular IRES. Cellular IRESs contain the short *cis*-elements that can base pair to the 18S rRNA. Therefore, these cellular IRESs can directly interact with 40S ribosomal subunit by base-pairing between itself and 18S rRNA and recruit 60S ribosomal subunit to start the translation.

The cellular IRESs can be roughly classified into two types based on the mechanisms of ribosome recruitment: type I IRESs interact with ribosomes through ITAFs that bound on the *cis*-elements, e.g. RNA binding motifs and N-6-methyladenosine (m^6^A) modification (Komar and Hatzoglou, 2011; Meyer et al., 2015; Yang et al., 2017; Figure 2A); whereas type II IRESs contain a short *cis*-element that pairs with 18S rRNA to recruit ribosomes (similar to Shine-Dalgarno sequences in bacteria) (Dresios et al., 2006; Figure 2B). Hundreds of studies have shown that different eukaryotic mRNAs contain certain regions with IRES activity, and a recent screen using an *in vivo* translation reporter has demonstrated that ~10% of mammalian mRNAs contain some elements to function as IRESs (Weingarten-Gabbay et al., 2016). However, the existence of cellular IRESs is still under debate (Kozak, 2005, 2007; Gilbert, 2010; Jackson, 2013), mainly because the systems to measure IRES activity are not reliable (discussed in detail later). In addition, the mechanisms of cellular IRESs are largely unclear compared to viral IRESs.

## IRES *trans*-acting factors

With the exception of Group I and Group II viral IRESs, most IRESs require the assistance of several ITAFs to recruit ribosomes for translation initiation. Almost all ITAFs are RNA binding proteins recruited by IRES elements to facilitate the ribosome assembly onto pre-mRNA (Figure 2). Most ITAFs were identified as nuclear proteins or proteins shuttling between the nucleus and cytoplasm, implying a crosstalk between translation and RNA transcription/processing. The La protein (La autoantigen) is the first ITAF discovered to promote the translation driven by PV IRES (Meerovitch et al., 1993). Many other proteins were later reported as ITAFs to promote IRES activity using *in vitro* or *in vivo* experiments (see the list of IRESs and bound ITAFs in IRESites: http://www.iresite.org; King et al., 2010; Mokrejs et al., 2010). A well-studied example is the polypyrimidine tract-binding protein (PTB) that can promote the activity for both viral and cellular IRESs. Interestingly, this protein is also involved in the regulation of mRNA splicing, stability, and transport (Sawicka et al., 2008), further suggesting the crosstalk between translation and RNA processing.

The exact mechanisms of how ITAFs facilitate IRES-dependent translation are largely unclear. It has been shown that some ITAFs (e.g. PTB) can function as RNA chaperones to remodel RNA structures around IRES, allowing for ribosome binding to the IRES. Alternatively, ITAFs can function as adaptor proteins to interact with ribosome or other translation IFs (Stoneley and Willis, 2004; King et al., 2010). The activity of most IRESs is fairly weak as judged by *in vitro* assays without the addition of ITAFs, which can increase the translation efficiency of cognate IRESs (Cobbold et al., 2008). Therefore, the efficiency of IRES-mediated translation generally varies in different cell types or tissues due to the expression level of ITAFs. Following this idea, a synthetic IRES containing binding sites for tissue- or cell type-specific ITAFs may be used to achieve tissue-specific protein production through IRES-dependent translation.

## Reporter system to measure IRES activity

The existence of IRESs in cellular mRNAs has been under debate for many years due to the lack of reliable evidence that translation initiation is indeed driven by these IRESs (Kozak, 2005, 2007; Gilbert, 2010; Jackson, 2013). To measure the activity of IRESs, various *in vitro* and *in vivo* translation systems were developed. In an *in vitro* translation system, the rabbit reticulocyte lysate or the translationally active HeLa cell extract is incubated with synthesized RNAs to examine their IRES activities. Such cell-free translation system is a powerful tool to determine the minimally required elements of the IRES; however, several cellular and viral IRESs do not have activity without additional ITAFs supplemented in this system (Brown and Ehrenfeld, 1979; Pickering et al., 2003). The *in vivo* systems, on the other hand, use various translation reporters that are transfected into cells to assay for the products of cap-independent translation. In this case, the ITAFs are provided by the cell, and thus the activities of IRESs may vary in different cell types.

The activities of most viral IRESs have been validated using both *in vitro* and *in vivo* systems, and in several cases there are structural evidences for the direct interaction between IRESs and ribosomes (Spahn et al., 2004; Costantino et al., 2008; Fernandez et al., 2014; Muhs et al., 2015). However, the activity of many cellular IRESs was only supported by limited evidence in *in vivo* systems, and most of cellular IRESs do not have the IRES activity as judged by *in vitro* system. Several reports have shown that the cellular IRESs require a `nuclear experience’ for their function, i.e. they can only drive translation in mRNAs that have been transcribed and spliced in the nucleus before being transported into the cytoplasm (Stoneley et al., 2000; Semler and Waterman, 2008). The mechanism behind the requirement of `nuclear experience’ is unclear. A possible reason is that the *in vitro* synthesized RNAs lack necessary modifications or nuclear RNA binding proteins, and thus the activity of most cellular IRESs can only be observed by using *in vivo* systems.

## Common concerns of the IRES reporters

**Figure 3:**
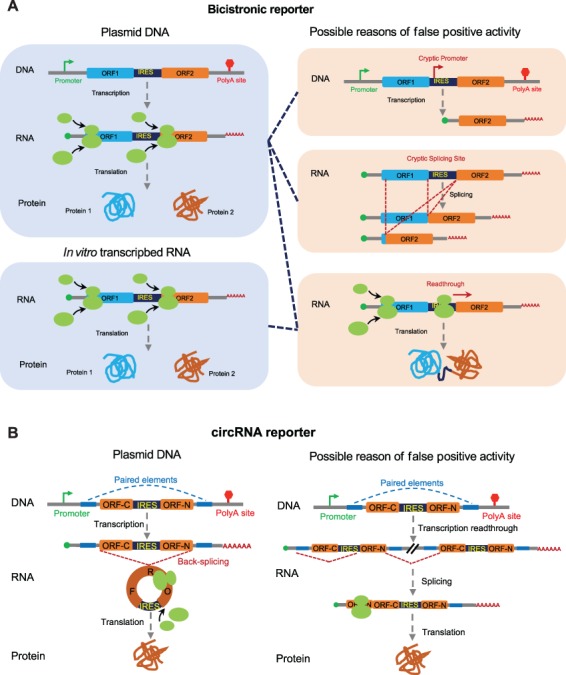
Reporter systems using in IRES study. (**A**) Bicistronic reporter system. The most popular reporter system is used to examine IRES activity. In the plasmid, two ORFs are inserted into downstream of the transcription promoter, and the IRES is inserted between these two ORFs. During translation, the first ORF is translated through cap-dependent translation whereas the second ORF is translated by cap-independent translation. This reporter can be *in vitro* transcribed into RNA and used for the *in vitro* or *in vivo* translation system (left panel). There are three reasons that may introduce the false positive activity of the ‘IRES’ sequence: first, the cryptic promoter activity; second, the cryptic splicing sites; and third, translation readthrough (right panel). (**B**) circRNA reporter system. The circRNA reporter is inserted into a single exon containing two split GFP ORFs in a reversed order and two flanking introns with complement elements. After transcription, the circRNA will be generated through back-splicing and then translated into protein through IRES-dependent translation (left panel). The transcription readthrough produces a very long linear RNA concatemer including two or more exons with the split GFP ORFs. This RNA concatemer may be spliced into the mature linear mRNA containing a whole ORF. Therefore, the transcription readthrough will introduce the false discovery activity of IRESs (right panel).

The most commonly used system to measure the IRES activity is the bicistronic translation reporter that contains two complete ORFs separated by an IRES within a single transcription unit (Figure 3A). The ORFs coding for renilla and firefly luciferases are often used in such reporters because their activities can be accurately quantified using a simple system with high sensitivity. After transcribed as a single mRNA, the first ORF is translated through cap-dependent translation, while the translation of the second ORF is initiated from the IRES. Such bicistronic reporters can be transfected into cells using a plasmid vector, or occasionally using *in vitro* transcribed RNA. As mentioned earlier, the *in vitro* synthesized RNAs lack `nuclear experience’, and thus it may not be efficiently translated. Although using plasmid vectors coding for bicistronic mRNAs can avoid this concern of `nuclear experience’, there are other problems that often induce artifacts in IRES activity.

Three common artifacts may contribute to the false discovery of IRESs by the bicistronic translation vectors: first, the DNA sequence for the `IRES’ may serve as a cryptic promoter to initiate alternative transcription, producing a new mRNA with only the second ORF; second, the transcribed mRNA may contain cryptic splicing sites that generate in-frame fusion ORF through alternative splicing; finally, the ribosomes may be able to restart the translation after finishing the first ORF, either by reading through the stop codon of the first ORF, shunting to the second ORF, or through re-initiation at the second ORF (Figure 3A).

Several careful control experiments are usually required to eliminate these artifacts. For the artifact caused by the promoter activity of `IRES’, the deletion of the first promoter or the siRNA knockdown targeting the first ORF should be performed as control experiments (Van Eden et al., 2004; Andreev et al., 2009). When the translation of the second ORF is not reduced in these conditions, it is likely that the observed `IRES’ activity is, in fact, a false positive caused by the internal promoter. For the artifacts caused by cryptic splicing sites, one should carry out the control experiments using RT-PCR to directly detect additional splicing isoforms produced from the bicistronic reporter. The `abnormal’ splicing isoform containing the second ORF should be further validated by sequencing (Van Eden et al., 2004). Finally, to examine the false positive IRESs due to translation re-initiation or ribosome readthrough, a hairpin structure is usually inserted before the start codon of the first ORF to inhibit cap-dependent translation. The IRES-driven translation of the second ORF should not be affected by such hairpin structure (i.e. independent of the first ORF), whereas the readthrough translation will be inhibited (Coldwell et al., 2000).

## New lessons learned from circular RNAs

A large amount of circular RNAs (circRNAs) have been identified in all known eukaryotes (Jeck et al., 2013; Memczak et al., 2013; Salzman et al., 2013; Ye et al., 2015), most of which are generated from linear pre-mRNA through back-splicing using canonical splicing machinery (Chen, 2016). Increasing evidences have demonstrated that circRNAs can function as mRNA to direct protein translation (Chen and Sarnow, 1995; Wang and Wang, 2015; Legnini et al., 2017; Pamudurti et al., 2017; Yang et al., 2017). Since circRNAs lack 5′ or 3′ end, the translation of circRNAs can only be initiated through cap-independent fashion, providing a reliable system to measure IRES activity.

The translatable circRNAs can be generated by the circularization of *in vitro* synthesized RNA using chemical or enzymatic ligation, or through a self-splicing Group I intron system (Petkovic and Muller, 2015). However, it is technically challenging to ensure that all *in vitro* synthesize RNAs in such preparation are actually circular. In addition, the *in vitro* synthesized RNAs will have the same concern of lacking `nuclear experience’. Alternatively, the circRNAs can be generated from plasmid vectors encoding pre-mRNAs that undergo back splicing, providing an *in vivo* system to measure IRES activity using circRNA translation. The typical circRNA reporter contains an IRES and split GFP sequences that can be joined together by precise back splicing (Wang and Wang, 2015; Yang et al., 2017; Yang and Wang, 2018; Figure 3B). Since the circRNA can only be translated through cap-independent fashion, this system will be able to avoid the false discovery of IRESs that occurred in linear RNAs (e.g. cryptic promoters, cryptic splicing sites, and translation readthrough). The only false positive scenario could be introduced by transcription readthrough, which produces a very long linear RNA concatemer including two or more exons with the split GFP ORFs. This artifact can be easily eliminated by linearizing the plasmid before transfection (Wang and Wang, 2015).

Although circRNA is naturally suitable for the IRES activity measurement, there are several general rules that should be applied to ensure the specificity of this system. First, the ORF of the reporter gene should be divided into two fragments in reverse order, and thus the intact ORF can only be formed in circRNA. The introns flanking the circular exon usually contain complementary elements that can form base pairs to facilitate back-splicing (for the detailed design, see Yang and Wang, 2018). This design can reduce the artifacts caused by potential cryptic promoters, cryptic splicing sites, and translation readthrough. In addition, the linearized plasmid should be used to eliminate the false discovery that induced by transcription readthrough. Finally, when the candidate IRES does not contain the stop codon, the stop codon depleted reporter gene can be used to produce large protein concatemers through rolling cycle translation, which is probably the strongest evidence of cap-independent translation.

## Rethinking the number of protein-coding genes in the human genome

It is well accepted that only 1%–2% of the human genome codes for proteins, while the rest largely comprises regulatory elements (e.g. promoters, introns, and non-coding RNAs). However, the precise number of protein-coding genes in the human genome is still an unanswered fundamental question. Currently, 19986 genes are annotated as being protein-coding by the GENCODE project (GENCODE version 30), ~80% of which have been supported by mass-spectrometry evidence in ProteomicsDB (Frankish et al., 2018; Schmidt et al., 2018). The rest 20% of the annotated protein-coding genes (also referred as `missing proteome’) are resulted from either technical limitations in proteomic survey (e.g. small secreted proteins are difficult to be detected) or the genome mis-annotation (e.g. some protein-coding genes are in fact mis-annotated pseudogenes) (Wilhelm et al., 2014).

Conversely, there could also be unknown proteins translated from the regions outside of the annotated protein-coding genes, suggesting the existence of a ‘hidden proteome’. Increasing evidence demonstrated that various small peptides are translated from non-coding regions as judged by ribosome profiling (Ingolia et al., 2014; Ingolia, 2016) or by improved analysis of mass-spectrometry data (Oyama et al., 2004; Baerenfaller et al., 2008; Slavoff et al., 2013; Wilhelm et al., 2014). Some of these newly identified peptides are found to play important roles in the regulation of transcription (e.g. Pgc in fruitfly) or enzymatic activity (e.g. the MLN and DWORF that regulate SECRA activity in human). More examples and information of these peptides can be found in recent in-depth reviews (Slavoff et al., 2013; Cabrera-Quio et al., 2016).

The RNAs encoding hidden proteome were originally annotated as non-coding RNAs because they lack typical features of mRNA. For example, these RNAs usually have a long 5′ UTR and short ORF with low translation efficiency. The long 5′ UTRs usually contain multiple stop codons, indicating that the downstream ORFs are probably translated through cap-independent mechanism driven by IRESs. In this case, the frequency and location of the cellular IRESs in the transcriptome become a critical issue.

Previous studies have shown that 10% of the mRNA may contain the IRESs (Spriggs et al., 2008; Weingarten-Gabbay et al., 2016); however, the number of IRESs in non-coding RNAs or regions is still unknown. Interestingly, using circRNA reporter genes, it was reported that many short RNA elements containing m^6^A have IRES-like activity to drive the cap-independent translation of circRNAs (Yang et al., 2017). In addition, a recent unbiased screen of short sequences using circRNA translation reporter has also identified many short elements (<10 nt) with IRES-like activity to drive cap-independent translation (Fan et al., 2018). In fact, any fragment >50 nt is expected to contain an IRES-like short element by chance (Fan et al., 2018). Such surprising finding suggests that the requirement of IRESs is easy to fulfill, and thus many sequences can drive cap-independent translation, especially when the cap-dependent translation is inhibited under stress conditions. Analogous to the ‘pervasive transcription’ of the genome, the abundance of short IRES-like elements may suggest a ‘pervasive translation’ of the transcriptome (Ingolia et al., 2014). This finding also implies that multiple ORFs can be coded within a single mRNA, with some ORFs being translated internally through cap-independent translation. Similar to alternative splicing, this `alternative translation’ may serve as a new mechanism to increase the proteome complexity encoded by the human genome.

Cap-dependent translation becomes impaired under stress conditions (Spriggs et al., 2010; Ryoo and Vasudevan, 2017), therefore it is tempting to speculate that many of the novel ORFs might encode peptides with functions in cellular stress response pathways. In addition, since circRNAs do not have 5′ and 3′ ends, the circRNAs containing an infinite ORF may also be translated in a rolling cycle fashion to produce a large protein concatemer (Chen and Sarnow, 1995; Abe et al., 2015; Fan et al., 2018). Such large protein concatemers have a high tendency to form aggregates due to a large amount of repetitive sequences, and thus may be toxic to the cells. Additional research is required in the future to illustrate the scope of cap-independent translation and the biological functions of these new proteins translated from circRNAs or from alternative ORFs of mRNAs.

## Conclusion and perspective

The mechanism by which viral IRESs drive cap-independent translation is well understood; however, cellular IRESs still need more reliable evidences to illustrate their scope and mechanisms. The major issue of cellular IRESs is that their IRES activity is relatively weak when tested with the *in vitro* translation system, possibly due to the lack of necessary RNA modification and/or the accessory nuclear RNA binding proteins. Therefore, a reliable *in vivo* system will be critical to examine the activity of cellular IRESs. Based on the previous studies and our knowledge, the circRNA reporter system will be a useful tool to examine the activity of cellular IRESs. Several screen experiments using different *in vivo* systems suggest that IRES-driven translation is more prevalent than previously expected.

## Funding

This work is supported by the National Natural Science Foundation of China (31570823, 31661143031, and 31730110 to Z.W.; 91753135 and 31870814 to Y.Y.). Z.W. is also supported by the CAS Pioneer 100-Talent Program (type A). Y.Y. is also sponsored by the Youth Innovation Promotion Association CAS and Shanghai Science and Technology Committee Rising-Star Program (19QA1410500).

**Conflict of interest:** none declared.
